# Shedding Light on Vampires: The Phylogeny of Vampyrellid Amoebae Revisited

**DOI:** 10.1371/journal.pone.0031165

**Published:** 2012-02-15

**Authors:** Sebastian Hess, Nicole Sausen, Michael Melkonian

**Affiliations:** Cologne Biocenter, Botanical Institute, University of Cologne, Cologne, Germany; The University of Hong Kong, China

## Abstract

With the advent of molecular phylogenetic techniques the polyphyly of naked filose amoebae has been proven. They are interspersed in several supergroups of eukaryotes and most of them already found their place within the tree of life. Although the ‘vampire amoebae’ have attracted interest since the middle of the 19th century, the phylogenetic position and even the monophyly of this traditional group are still uncertain. In this study clonal co-cultures of eight algivorous vampyrellid amoebae and the respective food algae were established. Culture material was characterized morphologically and a molecular phylogeny was inferred using SSU rDNA sequence comparisons. We found that the limnetic, algivorous vampyrellid amoebae investigated in this study belong to a major clade within the Endomyxa Cavalier-Smith, 2002 (Cercozoa), grouping together with a few soil-dwelling taxa. They split into two robust clades, one containing species of the genus *Vampyrella* Cienkowski, 1865, the other containing the genus *Leptophrys* Hertwig & Lesser, 1874, together with terrestrial members. Supported by morphological data these clades are designated as the two families Vampyrellidae Zopf, 1885, and Leptophryidae fam. nov. Furthermore the order Vampyrellida West, 1901 was revised and now corresponds to the major vampyrellid clade within the Endomyxa, comprising the Vampyrellidae and Leptophryidae as well as several environmental sequences. In the light of the presented phylogenetic analyses morphological and ecological aspects, the feeding strategy and nutritional specialization within the vampyrellid amoebae are discussed.

## Introduction

The history of the ‘vampire amoebae’ traces back to the original description of the genus *Vampyrella* by Cienkowski about 150 years ago [1]. Cienkowski described naked, filose amoebae with a conspicuous red colour and slender pseudopodia. Their fascinating feeding behaviour, i.e. the extraction of algal cell contents after local perforation of the host cell wall, likely inspired the name of the genus. Initially, *Vampyrella* was placed into a new group of protists, termed ‘Monaden’, together with diverse organisms united by their predominantly parasitic life style and superficially similar life histories [1]. The alternation of trophic, motile cells with immotile division cysts (digestive cysts) as well as the ability of cells to form plasmodia, were thought to relate these organisms to the myxomycete slime molds [1], [2]. Based on the presence or absence of flagella, the ‘Monaden’ were further divided into ‘Monadineae zoosporeae’, comprising flagellate organisms, and ‘Monadineae tetraplastae’ (later ‘Monadineae azoosporeae’), in which the motile stage resembled an amoeba or heliozoon lacking flagella and to which *Vampyrella* was allocated together with the non-coloured *Nuclearia* Cienkowski, 1865 [1], [2]. Since their original description several amoebae supposedly related to the ‘core vampyrellids’ (*V. lateritia* Cienkowski, 1865, *V. pendula* Cienkowski, 1865, *V. vorax* Cienkowski, 1865) have been described including e.g. *V*. *gomphonematis* Haeckel, 1870 [3], *V*. *variabilis* Klein, 1882 [4] and *V*. *inermis* Klein, 1882 and the new genera *Leptophrys* Hertwig & Lesser, 1874 [5], *Hyalodiscus* Hertwig & Lesser, 1874, *Arachnula* Cienkowski, 1876 [6], *Gobiella* Cienkowski, 1881 [7], *Vampyrellidium* Zopf, 1885 [2], *Theratromyxa* Zwillenberg, 1952 [8], *Asterocaelum* Canter, 1973 [9], *Lateromyxa* Hülsmann, 1993 [10] and *Platyreta* Cavalier-Smith & Bass, 2008 [11]. Unfortunately, several of these taxa have been only incompletely described and important information about the morphology, life history (e.g. the presence or absence of digestive cysts) and food preferences of the cells is still lacking. Thus, the taxonomy of the vampyrellid amoebae is currently uncertain and even confusing.

Meanwhile, speculations about the systematic affiliation of the vampyrellid amoebae continued, resulting in changing opinions about the name and composition of the group. Although initially associated with myxomycetes, the ‘Actinophrys-like’ appearance of *Vampyrella* and *Nuclearia* due to their long radiating filopodia also led to proposals relating the vampyrellid amoebae to Heliozoa [12]–[16]. Alternatively they were placed in the Proteomyxa Lankester, 1885 (also Proteomyxida), an ill-defined group of amoeboid protists that could not be easily placed elsewhere [17]–[20]. Other authors recognized the vampyrellids as true filose amoebae [21]–[28] placing them into the Aconchulinia De Saedeleer, 1934, essentially naked filose amoebae. This taxon was subsequently split into the Cristidiscoidida Page, 1987 (Nucleariidae Cann & Page, 1979 and Pompholyxophryidae Page, 1987) and the Cristivesiculatida Page, 1987 (Vampyrellidae Zopf, 1885 and Arachnulidae Page, 1987) mainly differentiated by the structure of their mitochondrial cristae [21], [29], [30].

Phylogenetic analyses using molecular markers have dramatically changed our perception of the interrelationships among amoeboid protists in general, and filose amoebae in particular [11], [31]–[35]. Naked filose amoebae were shown to belong to at least two phyla: The nucleariid amoebae with non-granular filopodia are members of the Opisthokonta corroborating earlier ultrastructural studies [29], [30], [36], whereas filose amoebae with granular filopodia belong to the phylum Cercozoa [37]–[39], more specifically, the class Granofilosea within subphylum Filosa [11], [38]–[40]. So far only three terrestrial taxa of filose amoebae with non-granular filopodia, traditionally classified as vampyrellid amoebae, have been subjected to molecular phylogenetic analyses using SSU rDNA sequence comparisons. *Platyreta germanica*, an organism referred to as ‘*Arachnula impatiens*’, and *Theratromyxa weberi* all group in the Cercozoa, however, in a different subphylum (Endomyxa Cavalier-Smith, 2002) than the naked filose amoebae with granular filopodia [11]. The position of these terrestrial vampyrellids suggested a sister group relationship of the vampyrellid amoebae to a clade comprising the plant pathogenic Plasmodiophorida and the genus *Phagomyxa*, parasitizing diatoms [11], [41]. The three terrestrial vampyrellids investigated to date, however, do not reflect the broad diversity of vampyrellid amoebae, and thus the question of the monophyly of the group remained uncertain.

In this study single cell-derived cultures of the limnetic ‘core vampyrellids’ *Vampyrella lateritia*, *V. pendula* and *Leptophrys vorax* (Cienkowski, 1865) Zopf, 1885 have been established and used to study phylogenetic relationships among vampyrellid amoebae by SSU rDNA sequence comparisons. The findings shed light on the evolution of vampyrellid amoebae and further help to characterize this fascinating group of amoeboid protists.

## Materials and Methods

### Establishment and maintenance of cultures

Single trophozoites as well as digestive cysts of vampyrellid amoebae were isolated from natural samples and enrichment cultures into half strength Waris-H [42] containing either *Zygnema pseudogedeanum* (strain CCAC 0199), *Oedogonium stellatum* (strain CCAC 2231 B) or *Cylindrocystis brebissonii* (strain CCAC 0118). The isolated cells gave rise to eight co-cultures consisting of a single cell-derived vampyrellid population and the respective food alga listed in Table 1. These cultures were kept at 16°C with a photon fluence rate of 10–30 µmol m^−2^ s^−1^ in a 14/10 hr light/dark cycle. At regular intervals of two weeks the vampyrellids were subcultivated using fresh algal material grown in Waris-H under the same conditions. The algal strains and vampyrellid-algal co-cultures are available through the Culture Collection of Algae at the University of Cologne (CCAC) at http://www.ccac.uni-koeln.de/ (For strain numbers see Table 1).

**Table 1:** Investigated strains of vampire amoebae and corresponding data.

Morphospecies	Strain	Strain number CCAC	SSU rDNA accession	Sequence length (nt)	Food source in culture	Origin
*Vampyrella lateritia*	VL.01	NA	HE609040	1640	*Zygnema pseudogedeanum* (strain CCAC 0199)	Freshwater pond, Grube Cox, Bergisch Gladbach, Germany; N50°58′27.27″E7°8′46.14″
	VL.02	CCAC 3426 B	HE609034	1595	*Zygnema pseudogedeanum* (strain CCAC 0199)	Large puddle with *Spirogyra* sp., Dutzenthal, Germany; N49°35′19.95″E10°27′3.07″
*Vampyrella pendula*	VP.01	CCAC 3427 B	HE609035	1698	*Oedogonium stellatum* (strain CCAC 2231 B)	Fishpond, Monsau, Wiehl, Germany; N50°57′34.99″E7°35′15.06″
	VP.02	NA	HE609041	1552	*Oedogonium stellatum* (strain CCAC 2231 B)	Pond on the Flower island in Lake Constance, Germany; N47°42′10.45″E9°11′41.86″
*Leptophrys vorax*	LV.01	CCAC 3422 B	HE609036	1670	*Closterium cornu* (strain CCAC 1125)	Edge of pond, Heiliges Meer, Recke, Germany; N52°20′57.12″E7°37′44.45″
	LV.02	CCAC 3423 B	HE609037	1727	*Closterium cornu* (strain CCAC 1125)	Pond in old stone pit, Sengelbusch, Brüchermühle, Germany; N50°56′13″E7°37′25″
	LV.03	CCAC 3424 B	HE609038	1572	*Closterium cornu* (strain CCAC 1125)	The Botanical Garden of the University of Münster, Germany; N51°57′49″ E07°36′38″
	LV.04	CCAC 3425 B	HE609039	1607	*Closterium cornu* (strain CCAC 1125)	Sphagnum ponds of Simmelried, Hegne, Konstanz, Germany; N47°43′3.96″E9°5′37.50″

CCAC = Culture Collection of Algae at the University of Cologne.

NA = not available (deceased).

nt = nucleotides.

### Light microscopy

Light microscopical observations were made with an inverted cell culture microscope (CK×41, Olympus, Hamburg, Germany) equipped with phase contrast through the bottom of plastic petri dishes or with an upright microscope (Zeiss Standard WL, Germany) using immersion objectives and DIC optics for high resolution.

### DNA-Amplification, sequencing and sequence assembly

The nuclear SSU rDNA was amplified via polymerase chain reaction (PCR) [43] using the DreamTaq™ DNA Polymerase (Fermentas, St. Leon-Rot, Germany) according to the manufacturers' instructions except for the DNA template. To avoid amplification of algal DNA single trophozoites and digestive cysts were isolated from cultures with the aid of a micropipette and used for PCR, followed by a semi-nested re-amplification. After pipetting single cells into 10 µl distilled water in PCR reaction tubes they were immediately frozen in liquid nitrogen. Directly before starting the PCR the mastermix was added to the frozen cells to prevent enzymatic DNA degradation. For primary PCR a combination of the universal eukaryotic primers EAF3 and ITS055R was used, whereas the semi-nested re-amplification was performed with the primer BR instead of ITS055R [44]. For both reactions the following PCR protocol was used: an initial denaturation step (95°C for 180″) was followed by 30 cycles including denaturation (95°C for 45″), annealing (55°C for 60″) and elongation (72°C for 180″). PCR products which showed single clear bands by gel electrophoresis were purified using the Dynabeads M-280 Streptavidin system [45]. For sequencing the SequiTherm EXCEL II Long Read DNA Sequencing Kit (Biozym Diagnostik, Germany) and the fluorescent labelled primer combinations EAF3/SSU-920R and SSU-528F/SSU-BR were used [44]. Two partial and overlapping sequences of each strand were read out with a LI-COR DNA Sequencer (LI-COR Biosciences, Lincoln, USA) and assembled to the complete SSU rDNA sequence using the program AlignIR™ 2.0 (LI-COR Biosciences, Lincoln, USA) [46]. All generated sequences have been deposited in GenBank and are available under the accession numbers shown in Table 1.

### Alignments and phylogenetic analyses

The SSU rDNA sequences of the eight vampyrellid strains varied in length between 1552 and 1722 nucleotides (see Table 1). Together with 48 previously published endomyxan sequences and 25 sequences of filosan Cercozoa as outgroup, the sequences were manually aligned in SeaView 4.3.0 [47], [48] based on the SSU rRNA secondary structure [49]. Hypervariable gene regions were analysed on ‘The mfold Web Server’ [50] resulting in an increased number of unambiguously alignable positions. After exclusion of non-alignable sites, the dataset containing 81 sequences and 1640 characters was analysed with distance (NJ), maximum parsimony (MP), maximum likelihood (ML) and Bayesian methods. For all analyses based on an evolutionary model of sequence evolution the best fitting model with respect to the data as well as the model parameters were estimated by the program Modeltest 3.0 [51]. Thus the model GTR+I+Γ and the parameters (the latter estimated by RAxML and MrBayes itself) were used for NJ, ML and Bayesian inference. ML analyses were done using the PTHREADS version of RAxML 7.2.6 [52]. To determine the best tree topology 20 distinct ML trees were computed starting from 20 distinct randomized maximum parsimony starting trees. Distance analyses (Neighbour Joining) [53] as well as MP analyses were performed with the program PAUP 4b10-×86-linux [54] under the default options. For the Bayesian inference the program MrBayes 3.1.2 was used [55], [56]. The Bayesian analysis was performed with two Markov chain Monte Carlo (MCMC) chains with 5,000,000 generations under the default options with the covarion model and autocorrelation. Trees were sampled every 100 generations and the ‘burn-in’ (1,500,000 generations) was determined by the convergence criterion. The statistical support for branches was calculated by bootstrapping with 1000 replicates using NJ, MP and ML [57]. All supporting values including posterior probabilities (BI) were plotted onto the best ML topology, if the respective branches were present. For converting NEXUS tree files and graphical tree design TreeView 1.6.6 and Adobe Illustrator CS4 (Adobe Systems, München, Germany) were used respectively.

### Nomenclatural Acts

The electronic version of this document does not represent a published work according to the International Code of Zoological Nomenclature (ICZN), and hence the nomenclatural acts contained in the electronic version are not available under that Code from the electronic edition. Therefore, a separate edition of this document was produced by a method that assures numerous identical and durable copies, and those copies were simultaneously obtainable (from the publication date noted on the first page of this article) for the purpose of providing a public and permanent scientific record, in accordance with Article 8.1 of the Code. The separate print-only edition is available on request from PLoS by sending a request to PLoS ONE, 1160 Battery Street Suite 100, San Francisco, CA 94111, USA along with a cheque for $10 (to cover printing and postage) payable to “Public Library of Science”. Furthermore 15 hardcopies of this publication have been deposited in the following publicly accessible libraries: http://www.loc.gov/ (Library of Congress, USA), http://www.nla.gov.au/ (National Library of Australia), http://www.bn.br (Biblioteca Nacional, Brazil), http://nlc.nlc.gov.cn/ (National Library of China), http://www.bnf.fr/ (Bibliothèque nationale de France), http://www.ndl.go.jp/en/index.html (National Diet Library, Japan), http://www.rsl.ru/ (Russian State Library), http://www.bne.es (Biblioteca Nacional, Spain), http://portico.bl.uk/ (British Library, UK), http://www.naturkundemuseum-berlin.de/bibliothek.html (Museum für Naturkunde, Berlin, Germany), http://www.sil.si.edu/ (Smithsonian Institution, USA), http://www.lib.berkeley.edu/ (University of California Library, Berkeley), http://www.ub.uni-koeln.de/ (Universitäts- und Stadtbibliothek Köln, Germany), http://library.amnh.org/index.php (American Museum of Natural History Library, USA), http://www.nhm.ac.uk/research-curation/library/index.html (Natural History Museum, London, UK).

In addition, this published work and the nomenclatural acts it contains have been registered in ZooBank (http://zoobank.org/), the proposed online registration system for the ICZN. The ZooBank LSIDs (Life Science Identifiers) can be resolved and the associated information viewed through any standard web browser by appending the LSID to the prefix “http://zoobank.org/”. The LSID for this publication is: urn:lsid:zoobank.org:pub:63020640-1161-4804-80F4-145E357E9B5C.

The electronic version of this publication is also deposited in the following digital archives: PubMedCentral (http://www.pubmedcentral.nih.gov/) and LOCKSS (http://www.lockss.org/).

## Results

### Molecular phylogeny

**Figure 1:**
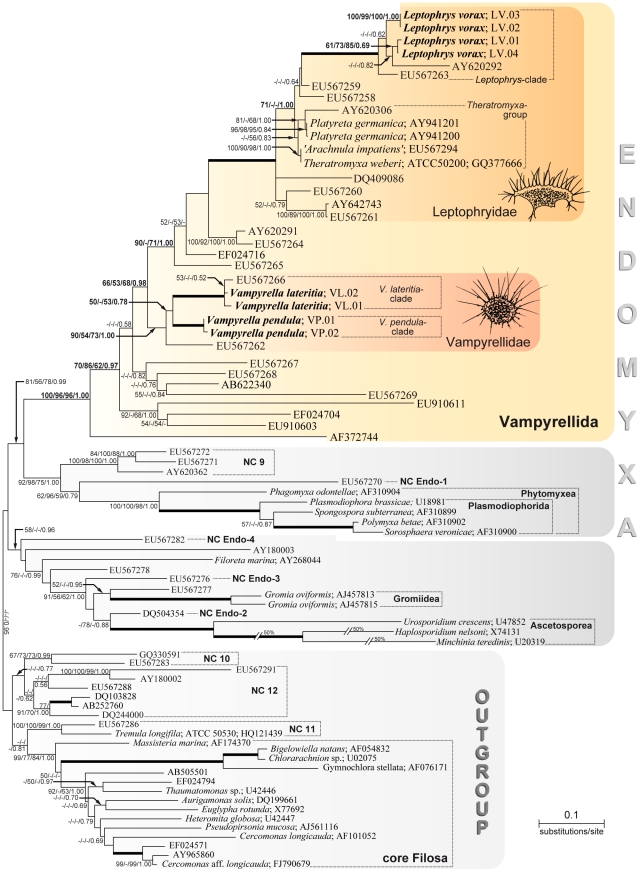
Reconstructed phylogeny of the Vampyrellida in a cercozoan context based on SSU rDNA sequence comparisons. Shown is the best maximum likelihood tree obtained by RAxML analyses of 81 sequences using 1640 aligned characters. The order Vampyrellida as well as the families Vampyrellidae and Leptophryidae are shaded in yellow/red colours, the remaining two deep-branching endomyxan clades in grey. The branch containing ‘Novel Clades’ 10–12 and some members of the ‘core Filosa’ are defined as outgroup. For previously published sequences taxonomic names and accession numbers are given. Sequences without taxonomic designations are environmental sequences and newly obtained sequences are combined with the strain designation only and are in bold (for accession numbers see Table 1). The support values of all methods applied are shown on the respective branches in the following order: ML/NJ/MP/BI. Support values less than 50% or 0.5 are not shown (−), whereas bold branches were maximally supported by all methods (100/100/100/1.00). Interrupted branches (//) show 50% of their original length. NC = ‘Novel Clade’ according to Bass & Cavalier-Smith (2004) and Bass et al. (2008) [11], [38].

The best ML tree shown in Figure 1 reveals the cercozoan subphylum Endomyxa and a filosan outgroup, the latter including the ‘Novel Clades’ 10–12 [58]. As in previous analyses using SSU rDNA sequence comparisons, the Endomyxa and some other basal branches within the Cercozoa are not supported [11], [58]. Within the Endomyxa three well known clades are resolved (highlighted in Fig. 1); (1) a not-well supported clade including the parasitic Ascetosporea Cavalier-Smith, 2002, the Gromiidea Cavalier-Smith, 2003, *Filoreta marina* Bass & Cavalier-Smith, 2008 as a representative of the Reticulosida Cavalier-Smith, 2003 and several environmental sequences representing ‘Novel Clades’ Endo-2, Endo-3 and Endo-4; (2) A well-supported clade comprising the class Phytomyxea Cavalier-Smith, 1993 (with Phagomyxida Cavalier-Smith, 1993 and Plasmodiophorida Cook, 1928) and sequences representing ‘Novel Clades’ Endo-1 and 9 as basal divergences; (3) The remaining large and well supported clade (100/96/96/1.00) named Vampyrellida here, reveals a high genetic diversity. It includes our new sequences of *Leptophrys* and *Vampyrella*, the previously published sequences of soil-dwelling vampyrellids [11], [41], along with environmental sequences.

Four *Leptophrys vorax* sequences together with two environmental sequences form a maximally supported clade, termed here ‘*Leptophrys*-clade’. Within the *Leptophrys*-clade the culture-derived sequences of the morphospecies *Leptophrys vorax* split into two distinct lineages containing strains LV.01 plus LV.04, and LV.02 plus LV.03, respectively (Fig. 1). The *Leptophrys*-clade in turn is part of a larger, maximally supported clade that also includes the previously investigated taxa ‘*Arachnula impatiens*’, *Platyreta germanica* and *Theratromyxa weberi*, and several environmental sequences. This clade has been referred to as the Vampyrellidae Zopf, 1885 by Bass et al. [11] but it is here described as Leptophryidae fam. nov. (see Taxonomic Revision, below). The sequenced strains of *Vampyrella* spp. are located outside the Leptophryidae in a more basal position robustly separated from the Leptophryidae by four environmental sequences (90/-/71/1.00). The four *Vampyrella* strains form an independent and well supported clade together with two environmental sequences (90/54/73/1.00), here designated as Vampyrellidae. The Vampyrellidae split into two lineages representing the morphospecies *Vampyrella lateritia* and *V. pendula*, each represented by two closely related culture-derived sequences (Fig. 1). One of the two environmental sequences (EU567262) within the Vampyrellidae is more distant from the two *Vampyrella* species, while the other (EU567266) is very similar to *V. lateritia*. The remaining branches of the endomyxan subclade Vampyrellida consist entirely of environmental sequences. Especially the long-branch environmental sequences in the more basal position within Vampyrellida are indicative of the need to increase taxon sampling of this group in the SSU rDNA database.

### Morphological identification

Using the original descriptions and additional published information, it was possible to assign the investigated vampyrellid strains to previously described taxa.

### *Vampyrella lateritia* (Fresenius, 1856) Leidy, 1879 (Fig. 2)

**Figure 2:**
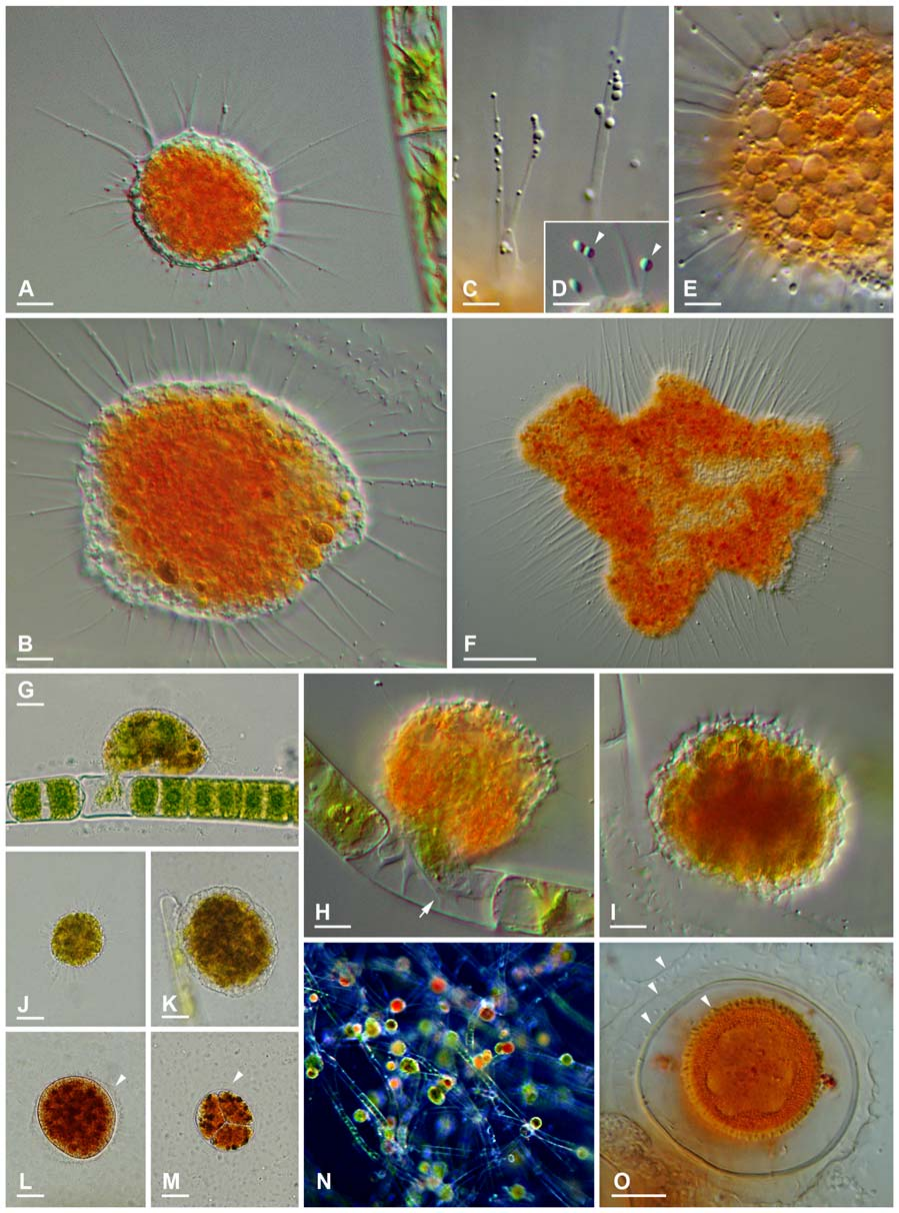
Life history stages and morphological traits of *Vampyrella lateritia*. **2A.** Advancing trophozoite. DIC. **2B.** Large trophozoite likely a result from cell fusions. DIC. **2C.** membranosomes moving along the pseudopodia. DIC. **2D.** ‘pin-like’ pseudopodia, arrowheads: membranosomes. DIC. **2E.** Numerous vacuoles in the cell periphery. **2F.** Large, bulky plasmodium. DIC. **2G.** Attached *Vampyrella* cell ingesting algal cell content. Brightfield. **2H.** Ingestion pseudopodium (arrow) emptying an algal cell. DIC. **2I.** Immobile *Vampyrella* cell with retracted pseudopodia and spiny surface turning into digestive cyst. DIC. **2J.** Green motile cell after food uptake. Brightfield. **2K.** Early digestive cyst stage with greenish contents. Brightfield. **2L.** Mature digestive cyst with delicate outer cyst envelope (arrowhead). Brightfield. **2M.** Digestive cyst with progeny after cell divisions in characteristic arrangement, arrowhead: outer cyst envelope. Brightfield. **2N.** Digestive cysts at various stages in different colours. Dark field. **2O.** Resting cyst with four envelopes (arrowheads). DIC. **Scale bars:** 2A, B, H, I, O = 10 µm; 2C–E = 5 µm; 2F = 50 µm; 2G, J–M = 20 µm.

Both strains (VL.01, VL.02) resemble the organisms described by Cienkowski (1865) as *V. spirogyrae* as well as those by Leidy (1879), Klein (1882) and Zopf (1885) as *V. lateritia* [1], [2], [4], [22].

**Observed life history stages.** Motile trophozoites, plasmodia, digestive cysts, resting cysts.

**Trophozoites.** Cells compact, often spherical, sometimes broader than long with respect to the direction of movement (Fig. 2A, B, J); size quite variable, young trophozoites about 25–40 µm, larger trophozoites (maybe derived from cell fusions) about 70 µm. Cell body with radiating pseudopodia, occasionally accumulating at the frontal edge during movement (Fig. 2A, B). Intense orange colouration of the central cell body, periphery and pseudopodia colourless. Cells float freely in the water column, or perform stalking, paddling movements. Pseudopodia long and thin, tapering, mostly unbranched; hyaloplasmatic; more or less equally distributed over the cell, sometimes in tufts. Numerous, highly refractive granules, about 0.5–1.5 µm in size (‘membranosomes’) moving along the pseudopodia (Fig. 2C), occasionally shooting rapidly out of the cell cortex connected by a thin cytoplasmic strand and subsequently retracted; the latter phenomenon results in the so-called ‘pin-like’ or ‘pinhead’ pseudopodia mentioned in the literature (Fig. 2D; arrowheads) [22], [59], [60]. Rarely formation of broad, claviform pseudopodia occurs; possibly related to pathological conditions. Occasionally numerous vacuoles present, predominantly in the peripheral cytoplasm (Fig. 2E), not always conspicuous at lower magnifications.

**Plasmodia.** Cells can fuse to large, deformed plasmodia showing the same structure and colour as the trophozoites (Fig. 2F); predominantly occurring in old cultures under food limitation.

**Digestive cysts.** Cyst outline roundish, elliptical, often slightly flattened towards the substrate when seen from the side; smallest roundish cysts about 35 µm in diameter, cysts more often slightly elliptical or oviform and ranging in size from 50–100 µm. Cysts with two cyst envelopes: outer envelope delicate, with even or slightly irregular surface, sometimes spiny outline (Fig. 2L, M; arrowheads); seems to resemble the cell outline before cyst formation and is likely formed at this stage (Fig. 2I, K). The inner cyst envelope is stronger than the outer, invariably with even surface; covering the cell body tightly until cell divisions commence (Fig. 2L, M). Young cyst stages greenish with roughly granulated content (Fig. 2K); during digestion turning orange-red (Fig. 2N); mature cysts with distinct food remnants as several brown conglomerates of various sizes (Fig. 2L). After internal cell division daughter cells (usually four) often visible in a typical tripartite arrangement (Fig. 2M); during hatching the food remnants are exocytosed and left behind in the empty cyst envelope.

**Resting cysts.** Seemingly four envelopes surrounding deep orange granular cyst content (Fig. 2O; arrowheads): the outermost envelope resembles the delicate, outer envelope of digestive cysts; with irregular, spiny outline. The next inner envelope is also very delicate, sometimes hardly visible; seems to be connected to the second innermost envelope by radial strand-like structures. The second innermost envelope is comparatively prominent, with even surface; maybe corresponding to the inner envelope of digestive cysts. The innermost envelope shows a warty surface; containing the spore content; diameter about 25 µm. Resting cysts only observed in strain VL.02.

**Feeding behaviour.** Trophozoites attach to an algal cell, often accompanied by retraction of the long pseudopodia as well as flattening of the cell body increasing the contact area (Fig. 2G). After several minutes the algal cell wall bursts locally and most of the extruded protoplast is quickly injected into a large food vacuole of the trophozoite (likely due to the turgor pressure). The remains of the disintegrated protoplast within the algal cell are engulfed by means of an ingestion pseudopodium (Fig. 2H; arrow). Several algal cells can be devoured in this way until the green amoeba enters the immobile digestive phase (Fig. 2J, K).

**Proven food organisms.** *Zygnema* spp. (strains CCAC 0199 and CCAC 0200), *Spirogyra* spp. (strains CCAC 1925 B, CCAC 3421); *V. lateritia* in the natural sample was feeding on *Spirogyra* sp.

### *Vampyrella pendula* Cienkowski, 1865 (Fig. 3)

**Figure 3:**
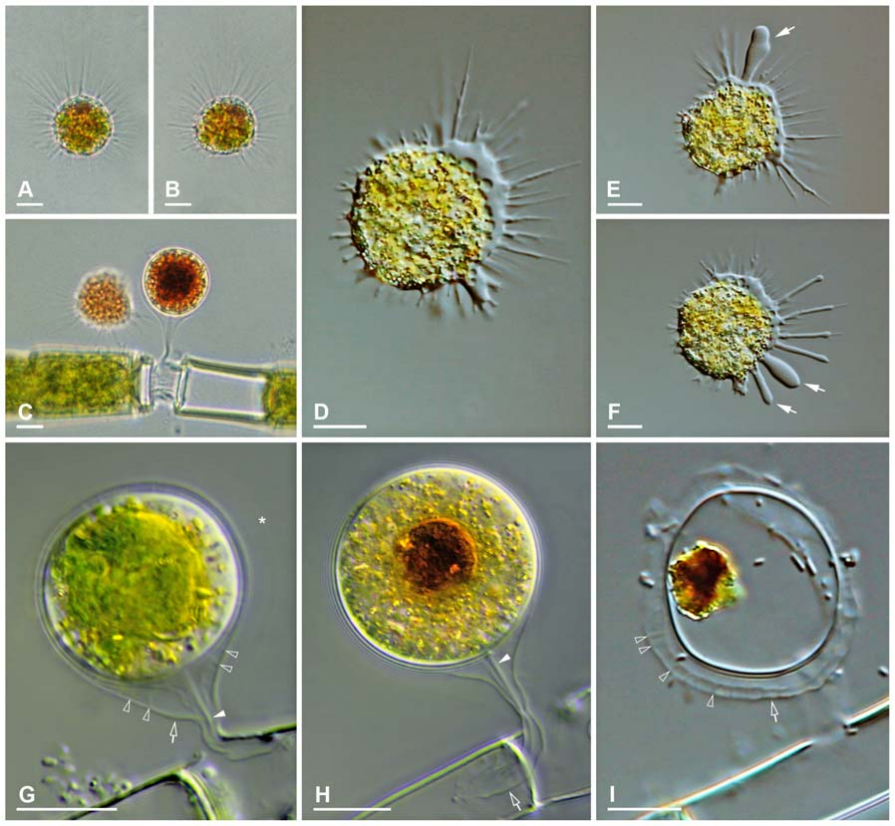
Trophozoites and digestive cysts of *Vampyrella pendula*. **3A & B.** Advancing trophozoites. Brightfield. **3C.** Trophozoite and digestive cyst on a filament of *Oedogonium*. Brightfield. **3D.** Trophozoite with clear pseudopodia lacking membranosomes. DIC. **3E & F.** Trophozoite producing claviform pseudopodia (arrows). DIC. **3G.** Early digestive cyst stage. DIC. **3H.** Mature digestive cyst. DIC. **3I.** Remaining digestive cyst envelopes with food remnant. DIC. Asterisk = very faint outer sheath, hollow arrows = outer cyst envelope, hollow arrowheads = delicate strands between outer and intermediate cyst envelope, arrowheads = central stand running from innermost cyst envelope through stalk. **Scale bars:** 10 µm.

Both strains (VP.01, VP.02) correspond to the organisms described by Cienkowski (1865), Klein (1882) and Zopf (1885) as *V. pendula* [1], [2], [4], [61]. The very similar organisms described as *V. inermis* by Klein presumably belong to the same species [4].

**Observed life stages.** Motile trophozoites, digestive cysts, resting cysts.

**Trophozoites.** Cells compact, often spherical, rarely deviating in shape (Fig. 3A, B); size of young trophozoites about 20–30 µm. Cell body with radiating pseudopodia, occasionally accumulating at the frontal edge during movement (Fig. 3D). Intense orange colouration of the central cell body, periphery and pseudopodia colourless (Fig. 3C, D). Cells float freely in the water column, or perform stalking, paddling movements. Pseudopodia very long (up to 50 µm) and thin, tapering, branched or unbranched; hyaloplasmatic; more or less equally distributed over the cell, sometimes in tufts. No membranosomes present on the pseudopodia, contrasting *V. lateritia* (Fig. 3D, E, F). Rarely formation of broad, claviform pseudopodia occurs, possibly related to pathological conditions (Fig. 3E, F; arrows).

**Plasmodia.** Cell fusion not yet observed.

**Digestive cysts.** Cyst outline spherical, or very slightly elliptical; size about 25 µm in diameter; attached to food alga with outer cyst envelope drawn out into a very characteristic hollow stalk (Fig. 3C, G, H). Three cyst envelopes: outer envelope delicate, with even surface; shape pyriform; distally attached within an empty algal cell and thus functioning as stalk (Fig. 3G, H, I; arrows). The next inner envelope delicate, sometimes hardly visible; seems to be connected to the outer by very faint strand-like structures (Fig. 3G, I; hollow arrowheads), resembling the delicate spines described for the resting cysts of *V. pendula* by Klein and Zopf [4], [61]. The innermost cyst envelope is comparatively prominent (Fig. 3I), with even surface; covering the cell body tightly before hatching; a hyaline strand seems to be connected to the innermost cyst envelope and runs within the two outer cyst envelopes to the distal attachment site of the cyst stalk (Fig. 3G, H; arrowheads). Young cyst stages with voluminous green content covered by colourless peripheral cytoplasm (Fig. 3G), exhibiting a very faint, transparent sheath (maybe mucilage) rarely visible in DIC (Fig. 3G; asterisk); during digestion the cytoplasm turns orange-red (Fig. 3C, H); mature cysts contain a central, brown food remnant, which is left behind in the empty cyst after hatching (Fig. 3H, I).

**Resting cysts.** Not yet observed in detail; roughly resembling those drawn by Klein (1882) [4].

**Feeding behaviour.** In general resembling *V. lateritia*; until now *V. pendula* not observed to feed successively on several algal cells.

**Proven food organisms.** *Oedogonium stellatum* (strain CCAC 2231 B), *Oedogonium cardiacum* (strain SAG B 575-1a); *V. pendula* in the natural sample was feeding on *Oedogonium* sp.

### *Leptophrys vorax* (Cienkowski, 1865) Zopf, 1885 (Figs. 4 & 5)

**Figure 4:**
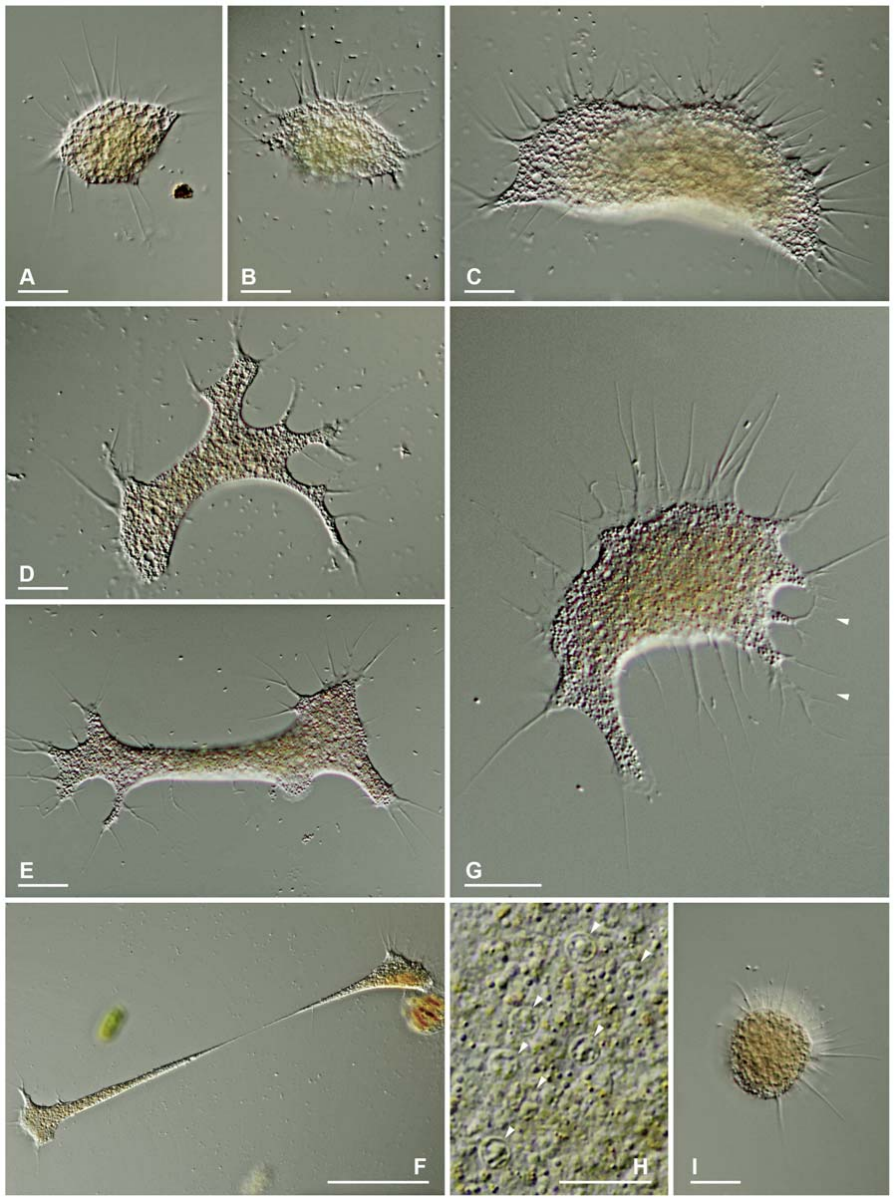
Trophozoites of *Leptophrys vorax* and their variability. **4A & B.** Small, compact trophozoites. DIC. **4C.** Advancing cell with fan-like outline. DIC. **4D.** Branched cell. DIC. **4E.** Elongated cell with several attachment sites. DIC. **4F.** Cell drawn out into thin cytoplasmic strand due to extending cell ends. DIC. **4G.** Advancing trophozoite showing clear pseudopodia emerging from a hyaline fringe, a tail-like posterior projection and dendritic pseudopodial structures (arrowheads). DIC **4H.** Vesicular nuclei in the cytoplasm of a dying individual (arrowheads). DIC. **4I.** Isodiametric floating form with radiating pseudopodia. DIC. **Scale bars:** 4A–E, 4G, I = 20 µm; 4F = 100 µm; 4H = 10 µm.

**Figure 5:**
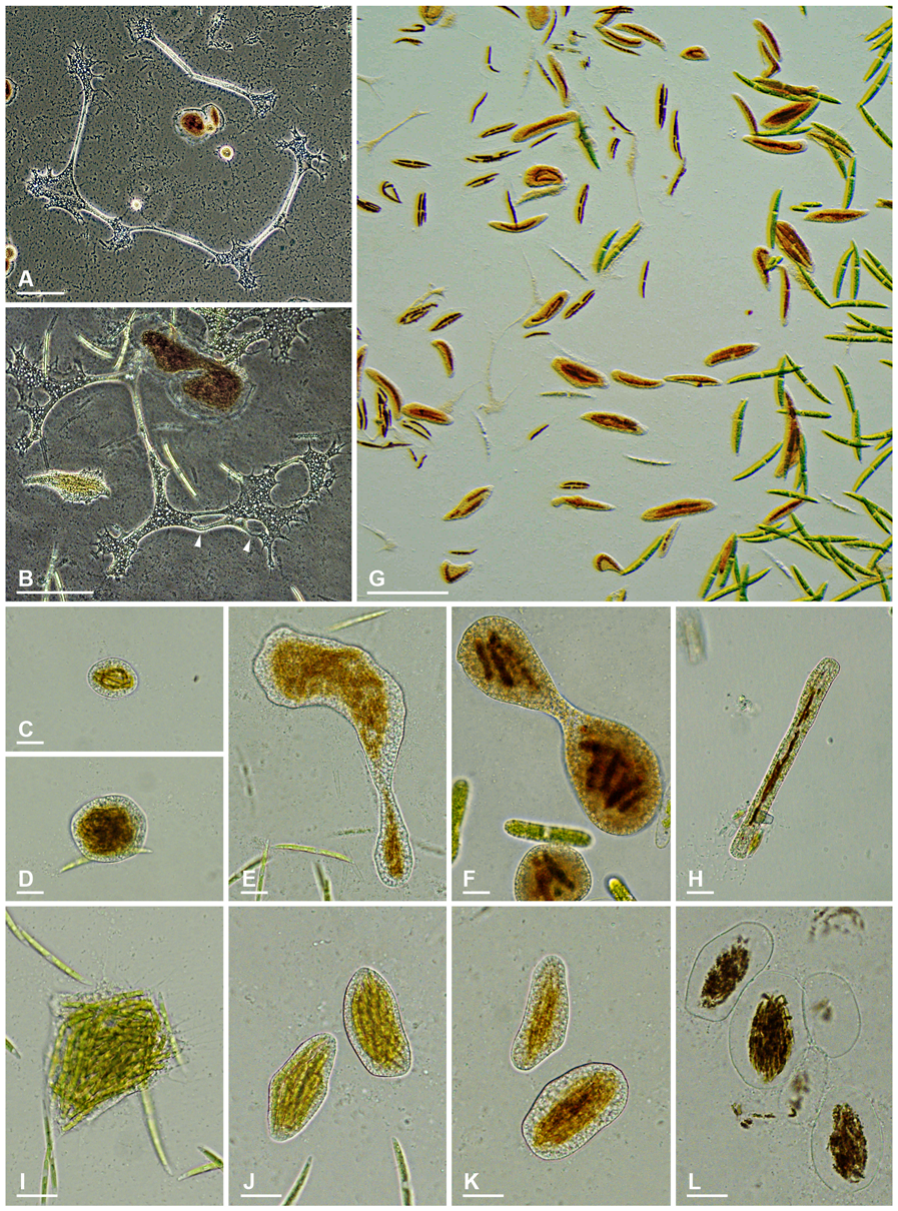
Plasmodia and digestive cysts of *Leptophrys vorax*. **5A.** Large extended Plasmodia. Phase contrast. **5B.** Plasmodium showing several anastomoses (arrowheads) and a normal sized trophozoite for comparison of the sizes. Phase contrast. **5C.** Small elliptical cyst. Brightfield. **5D.** Roundish cyst. Brightfield. **5E.** Irregular lobed cyst. Brightfield. **5F.** Dumbbell-shaped cyst. Brightfield. **5G.** Numerous digestive cysts in growing culture with *Closterium* sp. sometimes banana-shaped. Oblique illumination. **5H.** Slender cyst containing *Synedra* sp. Brightfield. **5I.** Moving trophozoite tightly packed with *Closterium* cells. Brightfield. **5J.** Early digestive cyst stage with greenish food inclusions. Brightfield. **5K.** Later digestive cyst stage with brownish food inclusions indicating the proceeding digestion. Brightfield. **5L.** Digestive cysts after hatching of the trophozoites just containing brown food remnants. Brightfield. **Scale bars:** 5A, B = 100 µm; 5C–F, H–L = 20 µm; 5G = 200 µm.

All strains (LV.01, LV.02, LV.03, LV.04) resemble the organisms described by Cienkowski (1865, 1876) [1], [6] and Klein (1882) [4] as *Vampyrella vorax*, those described as *Leptophrys elegans* and *L. cinerea* by Hertwig & Lesser (1874) [5] as well as the organisms designated as *L. vorax* by Zopf (1885) [2], [61]. The name *L. vorax* is adopted here (see Discussion).

**Observed life stages.** Motile trophozoites, plasmodia, digestive cysts.

**Trophozoites.** Cells extremely variable in shape and continuously changing the outline; usually flattened and spreading on surfaces. Small advancing individuals sometimes compact (Fig. 4A, B). Intermediate-sized cells can show a fan-like outline (Fig. 4C), can be branched into several arms (Fig. 4D), or elongate (Fig. 4E); sometimes drawn out to considerable length, then appearing as two cell bodies just connected via a thin, tense cytoplasmic stand (Fig. 4F). Due to adhesion on the substrate, moving organisms produce sticky tails, which stretch and finally are retracted (Fig. 4G). Size extremely variable, ranging from circa 40 µm to over one millimetre; clear distinction between single trophozoites and plasmodia not possible. Pseudopodia are predominantly produced at the edges of the cell, often originating from a hyaline and very delicate fringe of cytoplasm (Fig. 4G); sometimes in tufts. Pseudopodia long and thin, tapering, mostly unbranched; dendritic structures and anastomoses occur (Figs. 4G, 5B; arrowheads). No membranosomes present on the pseudopodia. Pseudopodial accumulation indicates direction of cell movement or cell extension (Fig. 4C, G), sometimes occurring on several sites in an individual (Fig. 4E, F). Colouration of the central cell body ranges from colourless to orange, depending on food source and contraction of the cell body; cell periphery and pseudopodia colourless (Fig. 4G). Cytoplasm often contains numerous vacuoles, several are contractile; vacuolation sometimes obscured due to numerous tiny refractive granules, but clearly visible at high magnification and in expanded individuals (Fig. 4A, C, D). At least two different populations of cytoplasmic granules distinguishable: colourless granules, possibly corresponding to the membranosomes of *Vampyrella*. Orange granules, possibly lipid droplets containing dissolved carotenoids of the prey. Numerous nuclei; very inconspicuous, often not visible in living individuals, but appearing as vesicular structures (about 3.5 µm in diameter) in squeezed dying cells (Fig. 4H, arrowheads); nuclei correspond to the data given by Zopf (1885) [61]. Cells move very smoothly over surfaces accompanied by the occasional retraction of the sticky posterior ends. When food is scarce trophozoites can transform into the isodiametric morphotype, detach and float in the water column. These compact cells, about 30 µm in diameter, resemble *Vampyrella* due to their radiating pseudopodia (Fig. 4I).

**Plasmodia.** Due to plasmodial organisation of unfused trophozoites, difficult to distinguish from the latter; very large plasmodia can exceed one millimetre (Fig. 5A, B).

**Digestive cysts.** Cysts vary greatly in size and shape depending of type and amount of prey. The smallest cysts are elliptical or roundish and about 30 µm in size (Fig. 5C, D), whereas large cysts can reach several hundred microns in length. Larger cysts can be elongated, irregular lobed (Fig. 5E), or dumbbell-shaped (Fig. 5F); when single large algal cells are engulfed, the cyst outline resembles the prey, as shown in case of banana-shaped cysts containing *Closterium* sp. (Fig. 5G) or very slender cysts containing *Synedra* sp. (Fig. 5H). The digestive cysts exhibit only one tight fitting cyst envelope, with even surface; corresponding to the inner cyst envelope of *Vampyrella*. Young stages reveal the green algal prey in the centre of the cell surrounded by colourless or slightly orange cytoplasm (Fig. 5J), in older stages the ingesta turned brown (Fig. 5K). During hatching of the trophozoites the food remnants are left behind in the empty cyst envelope (Fig. 5L). Although internal cell division occurs, the daughter cells occasionally fuse again outside of the cyst envelope during hatching.

**Resting cysts.** Not observed.

**Feeding behaviour.** Trophozoites engulf whole prey cells during their movement, often resulting in several food items collected in the cytoplasm of an individual (Fig. 5I). The occasionally observed change in colour of the prey indicates ongoing digestion before entering the immobile cyst stage.

**Proven food organisms.** *Cylindrocystis brebissonii* (strain CCAC 0118), *Closterium cornu* (strain CCAC 1125), *Closterium* sp. (strain CCAC 2687 B), *Planotaenium interruptum* (strain CCAC 0215), *Saccharomyces cerevisiae*, several diatoms and fragments of filamentous algae (observed in natural samples prior to isolation).

### Taxonomic Revisions and Diagnoses

The new results, more precisely the phylogenetic position of the genus *Vampyrella*, gave reason to revise the order Vampyrellida West, 1901 and to use it for the designation of the endomyxan clade containing the genera *Vampyrella*, *Leptophrys*, *Theratromyxa*, *Platyreta* and ‘*Arachnula*’. Previously, this clade was designated as ‘Novel Clade 8’ [38] and later tentatively as Aconchulinida De Saedeleer, 1934 [11]. An extensive literature research, however, suggested the adoption of the older and better defined ordinal name Vampyrellida for this clade. To establish a reasonable subdivision of the Vampyrellida the family Vampyrellidae Zopf, 1885 is emended and assigned to a monophyletic subgroup comprising all sequenced strains of the genus *Vampyrella*, including its type species, *V. lateritia*. For the morphologically similar genera *Leptophrys*, *Theratromyxa* and *Platyreta* the family Leptophryidae fam. nov. is erected. The taxonomic diagnoses are given below and detailed explanations can be found in the Discussion.

**Figure 6:**
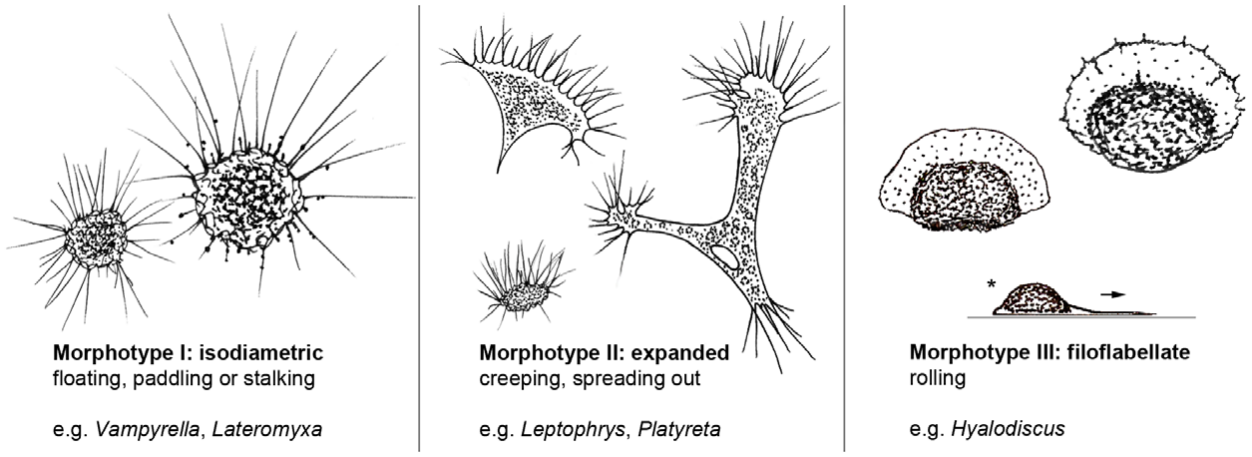
The three distinct morphotypes of vampyrellid amoebae. The shown morphotypes relate to the predominant morphology of locomotive trophozoites present in a growing vampyrellid population. Despite the existence of transitions from morphotypes II and III into a floating form resembling morphotype I (e.g. under food limitation) they serve well for describing the general appearance of the very variable vampyrellid taxa. Arrow = direction of movement, asterisk = side view.

**Order Vampyrellida West, 1901 emend.** Emended diagnosis: Exclusively heterotrophic, naked, amoeboid organisms; free-living in freshwater, soil or marine environments. Phagotrophic nutrition. Life cycle includes amoeboid, free moving trophozoites alternating with an obligatory digestive cyst, in which usually digestion and cell division take place; several taxa of the Vampyrellida can fuse to plasmodia and reach considerable sizes; sexual processes unknown. Cytoplasm often differentiated into a finely granular, sometimes highly vacuolated part (granuloplasm) and a structure-less hyaloplasm; the latter often surrounding the main cell body, but at least constituting the pseudopodia. All types of pseudopodia structure-less and without colour. Some Vampyrellida contain membranosomes in the cell periphery, sometimes migrating along the pseudopodia. Three major morphotypes of trophozoites distinguishable (Fig. 6):

1. **Isodiametric morphotype.** compact cells with more or less equally distributed, slender, filose pseudopodia sometimes emerging in tufts; cells float in the water column, perform paddling movements or stalk over surfaces.
2. **Expanded morphotype.** flattened and broadly attached cells, often spreading on surfaces; continuously changing their outline and therefore extremely variable in size and shape (e.g. irregular branched, elongate, anastomosing, network-forming); often bearing hyaloplasmatic fringes with radiating filose pseudopodia.
3. **Filoflabellate morphotype.** cells with broad, frontal lamellipodium consisting of clear hyaloplasm only exhibiting membranosomes, thus often appearing fan-shaped, sometimes the lamellipodium surrounds the whole cell outline; conspicuous filopodia absent, but often very delicate, filose subpseudopodia present on the ventral side (unpublished observations); main cell body in form of a granuloplasmatic hump; cells perform a characteristic rolling movement.

The order Vampyrellida can be phylogenetically defined as the largest well supported clade within Endomyxa including *Vampyrella*, *Leptophrys*, *Platyreta* and *Theratromyxa*, but excluding Phytomyxea.

**Genera included.** *Gobiella, Hyalodiscus, Lateromyxa, Leptophrys, Platyreta, Theratromyxa, Vampyrella*

**Family Vampyrellidae Zopf, 1885 emend.** Emended diagnosis: Limnetic Vampyrellida; predominantly showing the isodiametric morphotype in the trophic phase. Exhibiting straight, tapering and occasionally branching filose pseudopodia. Trophozoites of known members perforate algal cell walls (e.g. of Zygnematophyceae or Chlorophyceae) and phagocytose only the cell contents; thus seemingly strictly specialized on the food organisms. Cells show intense orange colour due to the algal diet, except directly after phagocytosis when the cells are green. Digestive cysts isodiametric, sometimes slightly flattened; at least two distinct cyst envelopes present; a delicate outer cyst envelope usually attaches the cyst to a substrate (e.g. the food alga), occasionally forming a stalk. The family can be phylogenetically defined as the largest well supported clade within the Vampyrellida including *Vampyrella*, but excluding *Leptophrys* (previously described as *Vampyrella vorax*).

Genera included: *Vampyrella*

**Family Leptophryidae Hess, Sausen et Melkonian fam. nov.** urn:lsid:zoobank.org:act:A1E44C59-7C57-49C4-BE3F-A3E1DC5B2E84

**Diagnosis.** Limnetic or terrestrial Vampyrellida; predominantly exhibiting the expanded morphotype. Cells spreading on surfaces in the trophic phase, continuously changing their outline and therefore extremely variable in size and shape (e.g. irregular branched, elongate, anastomosing, network-forming). Pseudopodia thin, tapering, occasionally ensiform; sometimes branching or dendritic; often emerging from hyaloplasmatic fringes at the cell margins; sometimes in tufts. Cells move by incessant creeping. Food items are engulfed as a whole (e.g. unicellular or colonial algae, fragments of algal filaments, fungal spores, yeast cells and small metazoans) or opened by local perforation of the cell wall (e.g. fungal conidia by *Platyreta germanica*). Colour of granuloplasm varies with food source; colourless or pale, algivorous members occasionally show yellowish, brownish or orange tint. Size and shape of digestive cysts depend on the food source; cysts sometimes resembling the outline of the prey. Outer, delicate cyst envelope known for Vampyrellidae emend. not observed.

**Type genus.** *Leptophrys*

Other genera included: *Theratromyxa*, *Platyreta*

## Discussion

### Phylogenetic results improve vampyrellid taxonomy

Although the ‘vampire amoebae’ aroused the interest of numerous scientists during the last two centuries, their phylogeny remains uncertain due to the paucity of cultured representatives that could be studied in detail by modern methods. With the molecular phylogenetic analysis of eight strains of vampyrellid amoebae belonging to the three important founding taxa *Vampyrella lateritia*, *V. pendula* and *Leptophrys vorax* described by Cienkowski [1] we resolved some of this uncertainty. We provided evidence that the terrestrial taxa (*Theratromyxa*, *Platyreta* and ‘*Arachnula*’), the voracious freshwater forms (*Leptophrys*) and the highly specialized cell wall-perforating species of the genus *Vampyrella* share a monophyletic origin as previously suspected on the basis of similarities in their life histories [8], [10], [62]–[64]. To this well-supported endomyxan clade we assign the oldest ordinal name Vampyrellida West, 1901 instead of Aconchulinida De Saedeleer, 1934. In addition to its nomenclatural priority, the order Vampyrellida is based on the genus *Vampyrella*, whereas the latter order has had a confusing history and is based on the genus *Penardia* Cash, 1904 whose phylogenetic position has yet to be determined [23], [65]–[67]. Furthermore, the name Vampyrellida allowed the required emendation of the family Vampyrellidae Zopf, 1885 without interfering with the commonly used term ‘vampyrellid’ often applied to the whole group of Vampyrellida [59], [62], [63], [68]. It was further shown that the taxa investigated split into three distinct clades, which could be linked to the two families Vampyrellidae Zopf, 1885 emend. and Leptophryidae fam. nov. Their known members differ significantly in morphology and behaviour.

The Vampyrellidae are now restricted to members of the type genus *Vampyrella* thus changing its concept drastically. In comparison with other higher rank taxa within Endomyxa and considering the high sequence diversity inside the newly defined order Vampyrellida, it seems appropriate to assign family status to the Vampyrellidae and Leptophryidae as defined here [11]. The two important founding species of the genus *Vampyrella*, *V. lateritia* and *V. pendula*, exhibit considerable sequence diversity and represent two lineages, which conforms to the morphological species concept used for these species. It is very likely that the Vampyrellidae will expand in the future when additional species of *Vampyrella* will be subjected to molecular phylogenetic analyses. However, some of the previously described *Vampyrella* species may turn out to group outside the Vampyrellidae or even outside the Cercozoa as indicated by fundamental differences in morphology and life histories (e.g. *V. incolor*, *V. radiosa*) [69].

The new family Leptophryidae comprises the limnetic genus *Leptophrys* and the terrestrial genera *Theratromyxa* and *Platyreta* as well as an organism named ‘*Arachnula impatiens*’. Whereas the latter are closely related, *Leptophrys* belongs to a distinct lineage (see Results). Because of the extreme morphological variability of the trophozoites and related problems with species identification, the genus *Leptophrys* as well as the soil-dwelling vampyrellids have had a confusing taxonomic history, which is briefly summarized in the following paragraph.

### The true identity of the genus *Leptophrys*

In his first publication including vampyrellid amoebae Cienkowski (1865) described *Vampyrella vorax* which shared the same life history with the two other species of the newly established genus. The major difference was the engulfment of whole algal cells (diatoms, euglenids, desmids) [1]. In 1874, Hertwig & Lesser published observations on trophozoites that were very similar to those of *V. vorax*, but exhibited a highly vacuolated cytoplasm and the presence of nuclei which had previously not been observed in *Vampyrella*. Because of these characteristics the new genus *Leptophrys* was created with the two species *L. elegans* and *L. cinerea*, which differed only in the colour of the cytoplasm from each other [5]. A reinvestigation of *V. vorax* by Cienkowski proved that the colour of its cytoplasm was dependent on its nutritional status and the type of food engulfed. Furthermore, he showed the presence of highly vacuolated stages in the life history of *V. vorax* [6]. Cienkowski and several later investigators thus rejected the distinguishing criteria that Hertwig & Lesser used for *Vampyrella* and *Leptophrys* as well as for the two *Leptophrys* species [2], [4], [64], [70]. Nevertheless, Zopf retained the genus *Leptophrys* and transferred *V. vorax* to *Leptophrys* as *L. vorax* [2]. Zopf extensively investigated the nuclei of several vampyrellid amoebae by staining techniques and used the observed multiplicity of nuclei in *L. vorax* together with the assumed absence of contractile vacuoles as well as the voracious method of food uptake as distinguishing characters of *Leptophrys* against *Vampyrella*, which was thought to be uninucleate [2], [61]. Although Hoogenraad discussed the history of the genus *Leptophrys* in detail and knew the work of Zopf he retained *Vampyrella vorax* and suggested to combine *L. elegans* and *L. cinerea* under the name *Leptophrys elegans* [64]. This is likely the reason why Page used the name *L. elegans* instead of *L. vorax* for a species, which he placed into the Nucleariidae due to the voracious food uptake which these organisms have in common [21]. Röpstorf et al., in contrast, regarded the formation of a digestive cyst and some ultrastructural features (polyhelical bodies and membranous, electron-dense bodies) as apomorphic characters of the vampyrellid amoebae including *L. vorax* [63]. Furthermore, ultrastructural differences with respect to the shape of the nuclei in interphase and the behaviour of the nuclear envelope during mitosis led Röpstorf et al. to suggest that *Leptophrys* is more distantly related, and perhaps in a sister position to all other vampyrellid genera [63]. Because of all these contradictory, sometimes doubtful, findings concerning the organisms placed in the genus *Leptophrys*, the genus finally found its place within the ‘protists without contemporary identity’ [71]. As shown here, four strains of vampyrellid amoebae (LV.01–LV.04), which corresponded well with the morphological descriptions of *Vampyrella vorax* and *Leptophrys* spp., constituted a separate clade distant from *V. lateritia* and *V. pendula* within the Vampyrellida. Due to their specific phylogenetic position, the different life style and habitat, confusion with the morphologically similar terrestrial taxa can be excluded. For these reasons separation of *Leptophrys vorax* from *Vampyrella* at the genus (and family) level is fully justified. Although the creation of the genus *Leptophrys* by Hertwig & Lesser as well as its usage by Zopf were apparently not based on sound diagnostic characters (vacuolation, colour, number of nuclei, presence of contractile vacuoles), we prefer to use this generic name for the strains investigated in this study to link these organisms with the matching morphological descriptions of *Vampyrella vorax* and *Leptophrys*. Interestingly, the four *Leptophrys* strains split into two distinct lineages in the SSU rDNA phylogeny. This indicates significant genetic divergence among the morphologically indistinguishable strains and may lead to the establishment of several taxa at species rank in the future. We refrain from formal designations of such taxa here, until the morphological plasticity, ultrastructure and food spectrum of the strains have been studied in detail.

### Vampyrellids from soil and what we know about them

The three vampyrellid taxa isolated from terrestrial samples have been described as *Theratromyxa weberi*, *Platyreta germanica* and ‘*Arachnula impatiens*’ [8], [11]. They do not appear to feed predominantly on algae but on fungi or even metazoa present in soil and have been studied several times since the 1950s particularly because of their possible role in biological pest control [72], [73]. In 1952, *Theratromyxa weberi* was described by Zwillenberg as the sole species of a new genus which differed from previously known vampyrellid taxa in its ability to feed on nematodes [8]. Because of morphological similarities to other flattened, branching amoebae which form digestive cysts, it was placed into the family Vampyrellidae of the order Proteomyxa Lankester, 1885 [8]. Observations on an organism assigned to *T. weberi* by Sayre complemented our knowledge and led to a nicely presented life cycle of the organism [74]. *Platyreta germanica* was presumably discovered and studied first by Old in 1977 [75]. The organism drew attention because of its remarkable ability to perforate fungal spores, but was later also shown to feed on cyanobacteria, green algae, diatoms and even nematodes. This finding together with the superficial appearance of the trophozoites motivated Old & Darbyshire to name it *Arachnula impatiens* explicitly referring to observations made by Dobell on this taxon in 1913 [62], [76], [77]. Despite the fact that Dobell likely did not observe the real *Arachnula* but rather *Leptophrys*, further investigations of the terrestrial amoebae feeding on fungi by Pakzad revealed clear differences between the organism studied by Old and *Arachnula impatiens* as described by Cienkowski or Dobell [78]. This finally led to the erection of the new taxon *Platyreta germanica* [11]. Apart from *P. germanica* and *T. weberi* further isolates from soil indicate a considerable diversity of terrestrial vampyrellids with a wide geographical distribution [59], [68], [75], [79], [80]. Anderson & Patrick, for example, investigated different mycophagous vampyrellids, which produced at least three different types of holes in the cell walls of the conidia of *Cochliobolus sativus* [59]. Other isolates from the USA and Japan turned out to feed on fungal hyphae, too [68], [80]. In general, the assignment of taxonomic names such as *V. lateritia* or *V. vorax* to these isolates must be taken with reservation. Even if some morphological traits are similar, the names of limnetic taxa which drastically differ in their habitat and food source should not be used for terrestrial isolates without further investigations. Unfortunately all living material of the previously investigated organisms was lost. Regarding the identity of the organism within the *Theratromyxa*-group named ‘*Arachnula impatiens*’ the data are quite sparse. The name is applied to a poorly studied organism, which was never in stable culture [11]. The description provided by Bass et al. was exclusively based on trophozoites and thus lacks important information regarding other life history stages. Furthermore, there is no information about the main distinguishing character, that led Cienkowski to separate this taxon from other vampyrellid amoebae: The described twitchy movements as well as the easily recognizable granular streaming within the pseudopodia referred to in the original description of *A. impatiens* have not been reported for any other known member of the *Theratromyxa*-group or even other vampyrellids [6], [8], [78]. Interestingly, Anderson & Patrick investigated an isolate morphologically resembling *T. weberi* but engulfing fungal spores and occasionally unicellular algae, named *Theratromyxa* sp. [59]. These observations are similar to those of Bass et al. [11] and gain more importance since the published SSU rDNA sequence of ‘*A. impatiens*’ is almost identical to that of *T. weberi*. These findings question the identity of ‘*A. impatiens*’ and the designation of this name to a member of the *Theratromyxa*-group. Furthermore, it should be noted that some other rhizopods such as *Penardia* Cash, 1904 and *Biomyxa* Leidy, 1875 display granular pseudopodia [21], [22], [24], [66]. Possibly, *Arachnula* is much more distantly related to the core vampyrellids than is often assumed.

### Morphological diversity of vampyrellid amoebae

Although all vampyrellids show considerable morphological variability in the trophic amoeboid stage it is possible to distinguish three major morphotypes which are predominantly present within a vampyrellid population (see Results and Fig. 6). Nevertheless, transitions from one morphotype to another occur, seemingly induced by environmental conditions. In *Theratromyxa weberi*, *Platyreta germanica* and *Leptophrys vorax*, all representing the expanded morphotype, a transition to small isodiametric cells with long radiating pseudopodia resembling *Vampyrella* has been observed [8], [78]. Regarding *Leptophrys* this transformation clearly depends on the availability of food and occurs only when the food source is exhausted. Consequently, the transition into the compact floating morphotype is likely a migrating strategy. Another peculiarity is the fusion of individual cells to large plasmodia and even network-like structures. In the case of *Leptophrys* such changes also appear in old cultures devoid of food, in which the size of the branched plasmodia may extend one millimetre in length. Formation of plasmodia by cell fusion perhaps allows formerly independent cells to scan a larger area for food and therefore increase the chance of survival for the resulting ‘superindividual’ at the expense of dispersal. As a consequence the predominant morphotype of a vampyrellid amoeba needs to be determined on the basis of several individuals under conducive conditions (e.g. enough food). Considering the morphotypes of the investigated taxa in relation to the presented molecular phylogeny, a separation of the taxa with the isodiametric morphotype from those predominantly showing the expanded morphotype is apparent. Indeed, this difference can be used to circumscribe the two families Vampyrellidae and Leptophryidae, although one has to take into account high intra-clonal variability and environmentally-induced morphological plasticity. As a result a detailed analysis based on cultures kept under controlled environmental conditions is essential for the description of taxa.

### Habitats and ecological distribution

*Platyreta*, *Theratromyxa* and ‘*Arachnula impatiens*’ have been isolated from terrestrial habitats. At least the first two taxa have their origin in agricultural fields and sandy soil respectively, not very similar to ponds and lakes where most of the limnetic taxa were found. The organism designated ‘*Arachnula impatiens*’ was isolated from a mixture of mosses, lichens and detritus collected near the shore of Lake Baikal, Siberia [11]. Interestingly, all known soil dwellers are closely related and according to the phylogenetic analysis presented here likely emerged from limnetic ancestors which migrated into terrestrial ecosystems and preyed on the organisms available. For this the omnivorous habit known from the Leptophryidae may have been an important prerequisite. Furthermore, their branched expanding morphotype could be much more beneficial for locomotion and the search for food in the porous soil environment. The *Vampyrella* species, in contrast, have all been found in wads of filamentous algae in ponds and larger puddles. The compact floating morphology of the trophozoites in *Vampyrella* likely enables cells to reach the floating food source in deep waters. In several vampyrellid taxa resting cysts have been described, but germination from these structures has not yet been observed in culture. In particular, the strain VL.02 regularly produces large numbers of resting cysts in older cultures which perhaps relates to the temporary habitat where it was found (a puddle). The majority of the environmental sequences within the Vampyrellida are of terrestrial and only rarely of limnetic origin. Despite an exhaustive search for sequences in the NCBI database none of the environmental sequences of vampyrellids identified originated from marine environments. Nevertheless, some vampyrellid amoebae such as *Vampyrella gomphonematis* and *Vampyrelloides roseus* Schepotieff, 1912 are known from marine sources [3], [81].

### Mechanisms of food acquisition and specificity

In several cases a quite strict specificity of certain vampyrellid taxa regarding their food sources was assumed and occasionally even used as an important taxonomic character. For example *Vampyrella lateritia* was described as exclusively feeding on *Spirogyra* [1]. Nevertheless, the strains of *V. lateritia* investigated here are able to feed on different algal strains belonging to the genera *Spirogyra* and *Zygnema* (see Results). Furthermore West observed *V. lateritia* to feed on *Mougeotia* [23] indicating a food preference rather for filamentous Zygnematophyceae (*Spirogyra*, *Mougeotia* and *Zygnema*) than for *Spirogyra* only. In the case of *Platyreta* the organism studied was designated as mycophagous, whereas in the original description of *Theratromyxa weberi* carnivory (catching, engulfing and digesting Metazoa; in this case nematodes) is one of the most important characters [8]. However, both, *Platyreta* and *Theratromyxa*, are also able to feed on certain algae and *Platyreta* even catches and digests nematodes [78]. These observations suggest caution in accepting the described food specificity of vampyrellid amoebae, especially, in cases when only one food source had been available/offered. Early investigators observed different individuals in a natural sample feeding on different food items as in the case of *L. vorax* [1], [2], [4], [5]. However, under these conditions the taxonomic identity of the individuals observed cannot be reliably established. In consequence, single cell-derived cultures are essential for feeding experiments before assigning nutritional preferences to specific taxa. Regarding the Leptophryidae all members exploit a broad range of food items; *L. vorax* is able to engulf diverse algae differing greatly in size and phylogenetic affiliation, yeast cells or motile heterotrophic protists [2], [4]. *Platyreta* and *Theratromyxa* show some signs of specialization towards certain food sources, the former grows significantly better when feeding on fungal spores than on nematodes and also the mechanism of nematode digestion differs from that used by *Theratromyxa* (pseudopodial invasion versus engulfment) [78]. In contrast to the Leptophryidae, *Vampyrella lateritia* and *V. pendula* seem to be much more selective in their food preferences, namely the Zygnematales (Zygnematophyceae, Streptophyta; *V. lateritia*) or the Oedogoniales (Chlorophyceae, Chlorophyta; *V. pendula*). Although the investigated strains of *V. lateritia* attack several different strains of the genera *Spirogyra* and *Zygnema*, trials to feed them on two different *Oedogonium* species failed. Vice versa, *V. pendula* never fed on the Zygnematales offered. This implies nutritional specialization resulting in niche separation of related vampyrellid taxa occupying the same habitat. Not only with respect to food source but also in the mechanisms of food acquisition and digestion, the vampyrellid amoebae display a fascinating range of diversity. Most famous is the local cell wall destruction resulting in a hole, which enables several members of *Vampyrella* and *Hyalodiscus* to feed exclusively on the protoplast of filamentous algae [10]. Another distinct strategy of opening the prey cell is by the production of annular depressions in cell walls resulting in the removal of a disk of cell wall material. This is observed in *Gobiella closterii* (Poisson & Mangenot, 1933) Röpstorf, Hülsmann & Hausmann, 1994 [82], [83] and also in *Platyreta* [78]. Members of the Leptophryidae evidently can engulf whole food items and indeed *Leptophrys* seems to digest algal cells entirely, thus resembling the typical strategy of food acquisition in amoeboid cells. However, the engulfment of food organisms should not necessarily be regarded as a plesiomorphic trait of the vampyrellids. Two terrestrial vampyrellids, tentatively named ‘*Vampyrella vorax*’ and ‘*Theratromyxa* sp.’, have been reported to engulf fungal conidia, subsequently perforating them within the food vacuole during the digestive cyst stage [59]. Based on the molecular phylogeny presented, a separation of the seemingly strictly algivorous vampyrellid amoebae (Vampyrellidae) from those with omnivorous tendencies (Leptophryidae) is apparent (see Results). Furthermore, the Vampyrellida show a remarkable diversification of food preferences, even among closely related taxa (*Theratromyxa*-group). The astonishing ability to produce holes in the cell walls of prey cells can be found in two separate groups (*Theratromyxa*-group within the Leptophryidae and Vampyrellidae) and could either represent a homoplasy or a plesiomorphic trait present in the common ancestor of the two families.

### Concluding remarks

With the first SSU rDNA sequences of *Leptophrys* and *Vampyrella* this study shed some light on the phylogeny of the vampyrellid amoebae. Results from the phylogenetic analyses and light microscopy of eight strains from these two genera, could be clearly interpreted and have led to taxonomic changes, i.e. the recognition of two families within the Vampyrellida West, namely the Vampyrellidae Zopf (emend) and the Leptophryidae (fam. nov.). Ecological observations on habitat and food preference support the phylogeny presented. The protocol we provide here to establish single-cell derived co-cultures of vampyrellid amoebae and their food organisms may help others to study vampyrellid amoebae under controlled experimental conditions, which are necessary for a correct identification and the description of novel taxa. Thus we hope to stimulate further research into the biology of the ‘vampire amoebae’, organisms that have filled researchers with enthusiasm during the last 150 years.
